# Profile of Patients with Primary Biliary Cholangitis and Evaluation of Response to Ursodeoxycholic Acid in a Romanian Center—Retrospective Study

**DOI:** 10.3390/jcm14228240

**Published:** 2025-11-20

**Authors:** Matei Mandea, Speranta M. Iacob, Mihaela C. Ghioca, Cristian Gheorghe, Liliana S. Gheorghe

**Affiliations:** 1 Department of Internal Medicine, Discipline of Gastroenterology and Hepatology, University of Medicine and Pharmacy Carol Davila, 050474 Bucharest, Romania; 2 Digestive Diseases and Liver Transplant Center, Fundeni Clinical Institute, 022328 Bucharest, Romania

**Keywords:** primary biliary cholangitis, ursodeoxycholic acid, UDCA response, GLOBE score, URS score, UK-PBC score, Paris II criteria

## Abstract

**Background**: Primary Biliary Cholangitis (PBC) is a chronic autoimmune liver disease, characterized by cholestasis and fibrosis. Ursodeoxycholic acid (UDCA) is the first-line treatment for normalizing markers, slowing progression, and improving outcomes. Various criteria, including Paris II, POISE, and Barcelona, along with scores like GLOBE, UK-PBC, and URS, help assess response and prognosis. The aim of our study was to evaluate the patient’s profile and assess treatment response to UDCA. **Methods:** We conducted a retrospective study of 276 patients diagnosed with PBC, evaluated between 2011 and 2024 at Fundeni Clinical Institute in Bucharest. Of these, 117 patients were assessed for UDCA response at 12 months. Demographic, clinical, and biochemical data were collected. Treatment response was evaluated using established criteria and prognostic scores. Logistic regression analysis was used to identify significant predictors of treatment response, while ROC curves assessed predictive capabilities for liver-related outcomes (LRO) and UDCA biochemical response. **Results:** The cohort primarily consisted of women (95.28%) with a mean age at diagnosis of 53.89 years (95% CI, 52.43–55.34). 40.5% of patients developed liver cirrhosis during follow-up. At 12 months, the response rates to UDCA therapy were 44.44%, 41.88%, and 52.14% according to the Paris II, POISE, and Barcelona criteria, respectively. Biochemical improvement was significant, particularly the reduction of alkaline phosphatase (ALP) below 1.67× the upper limit of normal, which was observed in 62.39% of patients. ROC analysis demonstrated robust predictive capabilities, with UK-PBC (AUROC 0.899) and GLOBE (AUROC 0.867) scores accurately predicting LRO. A lower age at diagnosis, higher ALP values at diagnosis, and absence of sp100 were factors associated with non-response. **Conclusions**: This study is one of the first detailed analyses of PBC patients in Romania, examining population-specific characteristics and UDCA response rates. Low response rates at 12 months highlight the need for longer follow-up and the exploration of second-line therapies.

## 1. Introduction

Primary Biliary Cholangitis (PBC), as defined by the guidelines of the European Association for the Study of the Liver (EASL), is a chronic, cholestatic, progressive liver disease with an autoimmune mechanism that, through progressive fibrosis, leads to liver cirrhosis with its complications [1,2]. The EASL [1] and the American Association for the Study of Liver Diseases (AASLD) [3] guidelines agree on the following PBC diagnostic criteria: (1) the evidence of biochemical cholestasis with persistent increase in serum alkaline phosphatase (ALP) in patients with normal ultrasound examination of the biliary tract; (2) the presence of antimitochondrial autoantibodies (AMA-M2), present in 80–90% of cases, or the PBC specific antinuclear autoantibodies (ANAs) gp210 or sp100; (3) histologic evidence of nonsuppurative obstructive cholangitis involving interlobular bile ducts [1,4,5]. At least two of these criteria must be met for the PBC diagnosis to be considered valid.

Data from epidemiological studies conducted in the United States (US) and Western Europe indicate that PBC predominantly affects women, in approximately 80–90% of cases. However, it is worth noting that males experience a more severe impairment in terms of the condition [4,6,7]. Recent European meta-analysis data indicate a prevalence of 22 per 100,000 people, whereas in the US, the reported prevalence is 40.9 per 100,000 people. The average age at diagnosis ranges from 40 to 60 years [8,9].

Patients affected by PBC may present non-specific symptoms at onset, the most significant being itch and fatigue [1,5]. Patients may associate signs and symptoms with other autoimmune diseases than PBC. These include autoimmune thyroid disease, systemic sclerosis, Sjögren’s syndrome, rheumatoid arthritis, and celiac disease. However, it is notable that these conditions do not significantly impact the progression of the disease [4,10,11]. However, PBC can be associated with other autoimmune liver diseases, which are classified as Variant Syndromes. The most common Variant Syndrome is PBC-Autoimmune Hepatitis (AIH), characterized by specific AIH autoantibodies, increased total IgG, and distinct changes in liver biopsy [12]. The variant syndrome PBC-Primary Sclerosing Cholangitis (PSC) is characterized by typical changes on magnetic resonance cholangiography (MRCP), including stenosis and dilatation of the intrahepatic and extrahepatic biliary tree. When the Variant Syndrome is present, treatment may target both diseases initially or focus on the most active disease first–either cholestasis syndrome or inflammatory liver activity [12,13]. A notable association in the disease’s progression, separate from autoimmune correlations, is bone metabolic disease, characterized by the development of osteopenia and osteoporosis. This phenomenon is linked to decreased vitamin D absorption and calcium deficiency [4].

As of today, ursodeoxycholic acid (UDCA) is the only drug approved as a first-line treatment for PBC and the only one recommended by international guidelines, with a standard dose of 13–15 mg/kg/day [1,3]. The drug was proven to be effective in reducing biochemical parameters altered in PBC (ALP, bilirubin, GGT, cholesterol, and IgM), as well as in slowing the progression of the disease and improving long-term outcomes and survival to a life expectancy similar to that of disease-free individuals [13,14,15].

Recently, the obeticholic acid (OCA) was recommended as a second line of therapy for PBC patients without response to UDCA. OCA is a selective farnesoid X receptor (FXR) agonist introduced in 2016, following the publication of the POISE trial, and was approved by the United States Food and Drug Administration (US FDA) and the European Medicines Agency (EMA) [16]. Subsequently, in 2024, the license was withdrawn by the EMA and questioned by the FDA; as a result, all patients in Romania discontinued the treatment [13,16,17].

In 2024, both the EMA and FDA approved two additional drugs as second-line therapies for patients with PBC who do not respond to or cannot tolerate UDCA. These drugs belong to the class of Peroxisome Proliferator-Activated Receptor Agonists (PPAR) [13,18].

These new therapeutic agents, Seladelpar and Elafibranor, showed significant results compared to placebo in the pivotal trials RESPONSE (Seladelpar) and ELATIVE (Elafibranor) [19]. The two drugs received approval from the Romanian Ministry of Health in 2025 for commercialization, but at the time of writing the article, no information was available about when the National Health Insurance House of Romania would reimburse the cost [20,21].

The primary endpoints in these new therapeutic trials were those established in the POISE trial for OCA (the POISE criteria), specifically ALP <1.67 times the upper limit of normal (ULN), with a reduction of at least 15% of the baseline ALP, and bilirubin normalization [16].

In addition to POISE criteria as an endpoint for treatment response, multiple definitions for response to therapy have previously been established, using ALP, total bilirubin (TB), aspartate aminotransferase (AST), or GGT level. Among these, the most used are the Paris II criteria (AST <1.5 times ULN, ALP <1.5 times ULN, and normal bilirubin level after 12 months of treatment) and the Barcelona criteria (decrease of >40% of ALP or normalization at 12 months) [1,14,22]. These sets of criteria assess response to treatment at 12 months after initiation. In addition to these, quantitative prognostic scores have been developed, including the Global Assessment of Liver Outcomes score for PBC (GLOBE), UK Primary Biliary Cholangitis score (UK-PBC), and UDCA Response Score (URS). They use laboratory parameters, including ALP, AST, age at diagnosis, TB, albumin (ALB), and platelets (PLT), to predict response to treatment (URS) or the occurrence of complications, such as liver failure, and the need for liver transplantation. For the GLOBE score, a cut-off value of <0.3, and for the URS, a cut-off value of ≥1.41 [14,23,24,25].

In this study, we conducted a retrospective analysis of patients with PBC who were followed at a high-volume referral center for liver diseases in Romania. The aim was to evaluate the patient profile and response to UDCA treatment using established criteria and prognostic scores.

## 2. Materials and Methods

### 2.1. Study Design and Period of Inclusion

This retrospective study evaluated 276 patients who underwent evaluation at our clinic (Digestive Diseases and Hepatic Transplant Centre, Fundeni Clinical Institute, Bucharest) between 2011 and 2024.

### 2.2. Inclusion and Exclusion Criteria

The entire cohort consisted of patients diagnosed with PBC evaluated between 2011 and 2024.

The inclusion criteria were: for analyzing paraclinical data at the start of UDCA treatment, patients diagnosed in the hospital between 2011 and 2024 were included. To evaluate treatment response, patients who had lab tests at both the start and at 12 months of treatment were selected.

The exclusion criteria included: presence of antibodies to PBC without cholestasis, post-liver transplant patients, patients with incomplete paraclinical data, and those diagnosed before 2011.

### 2.3. Variables and Endpoints

The variables extracted from the hospital’s database included patient history, associated autoimmune diseases, metabolic bone disease, age, sex, clinical outcomes (such as death, liver transplantation, and complications of liver disease), treatments used, date of diagnosis, and date of last evaluation (whether it was a final clinical assessment, death, or liver transplant).

The laboratory parameters included were PLT, AST, ALT, GGT, ALP, TB, INR, ALB, Cholesterol, Triglycerides, Creatinine, and Sodium, measured at the first evaluation, 12 months, and 24 months, depending on their availability.

The outcomes evaluated in this study were clinical, represented by liver transplantation, liver-related death, and complications of liver cirrhosis. Liver-related outcomes (LRO) were defined as a composite endpoint including liver transplantation, liver-related death, and decompensated cirrhosis events such as variceal bleeding, hepatic encephalopathy, refractory ascites, or jaundice. Demographic, clinical, and biochemical data were collected. The endpoints used to assess UDCA treatment response were defined by the criteria described in the introduction as Paris II, Barcelona, and POISE.

### 2.4. Quantitative Prognostic Index Scores Used

The quantitative prognostic scores used were GLOBE, UK-PBC, URS, FIB-4 index, and APRI. Their calculation formulas used were the following:

- GLOBE score = 0.044378 × age at start of UDCA therapy + 0.93982 × ln (TB times the upper limit of normal [ULN] at 1 year follow-up) + 0.335648 × ln (ALP × ULN at 1 year follow-up) − 2.266708 × ALB level × the lower limit of normal (LLN) at 1 year follow-up − 0.002581 × PLT count per 109/L at 1 year follow-up + 1.216865 [26,27];
- UK-PBC risk score = 1 − baseline survival function ∧exp(0.0287854 × [ALP baseline and after 12 months of therapy × ULN − 1.722136304] − 0.0422873 × [{(ALT where this was available, otherwise AST, baseline and after 12 months of therapy × ULN/10)^−1^} − 8.675729006] + 1.4199 × [ln{TB after 12 months of therapy × ULN/10} + 2.709607778] − 1.960303 × [ALB at baseline × LLN − 1.17673001] − 0.4161954 × [PL count at baseline × LLN − 1.873564875]) [26];
- UDCA Response Score (URS) = 0.77 + 0.60 × (√total bilirubin at diagnosis [× ULN]) − 1 − 2.73 × ln (ALP at diagnosis [×ULN]) + 0.35 × ln (ALT at diagnosis [×ULN]) + 0.03 × age [yr] − 0.15 × (time from diagnosis to the start of treatment [yr]) − 0.56 × (change in ALP concentration from diagnosis to the start of treatment [×ULN]) [28];
- AST-to-Platelet Ratio Index (APRI) = (AST × ULN)/PLT × 100 [24];
- Fibrosis-4 index (FIB-4 index) = (Age × AST)/(PLT × √(ALT)) [29];

### 2.5. Statistical Analysis

Continuous variables with a normal distribution are reported as mean ± standard deviation (SD), while those with a non-normal distribution are presented as median and interquartile range (IQR). To determine whether to use parametric or non-parametric tests, the normality of the continuous variables was assessed using the Shapiro–Wilk test. Comparisons between groups were performed using the *t*-test or Mann–Whitney U test, as appropriate. Categorical variables, summarized as percentages, were compared using the Chi-square test.

Univariate analysis was conducted for clinical data, laboratory parameters, as well as the GLOBE, URS, and UK-PBC 5-year scores. Variables with a *p*-value < 0.05 were entered into the multivariate logistic regression model. Results are reported as odds ratios (ORs) with 95% confidence intervals (CIs), and statistical significance was set at *p* < 0.05.

The predictive performance of each risk score was evaluated by calculating the area under the receiver operating characteristic curve (AUROC) and its corresponding 95% CI.

Survival curves were generated using the Kaplan–Meier method, based on the total duration of each patient’s disease progression and liver transplantation as the clinical endpoint. The comparison of the curves, for treatment response, was tested using the log-rank test. The number of patients at risk, indicated below the graph, was assessed at 12 months, corresponding to the evaluation of treatment response.

Statistical analysis was performed using SPSS (version 26.0; IBM Corp., Armonk, NY, USA), MedCalc (version 22; MedCalc Software Ltd., Ostend, Belgium), and DataTAB (Online Statistics Calculator, DataTab e.U. Graz, Austria; URL https://datatab.net/; Access date 1 October 2025).

## 3. Results

### 3.1. Population

**Figure 1:**
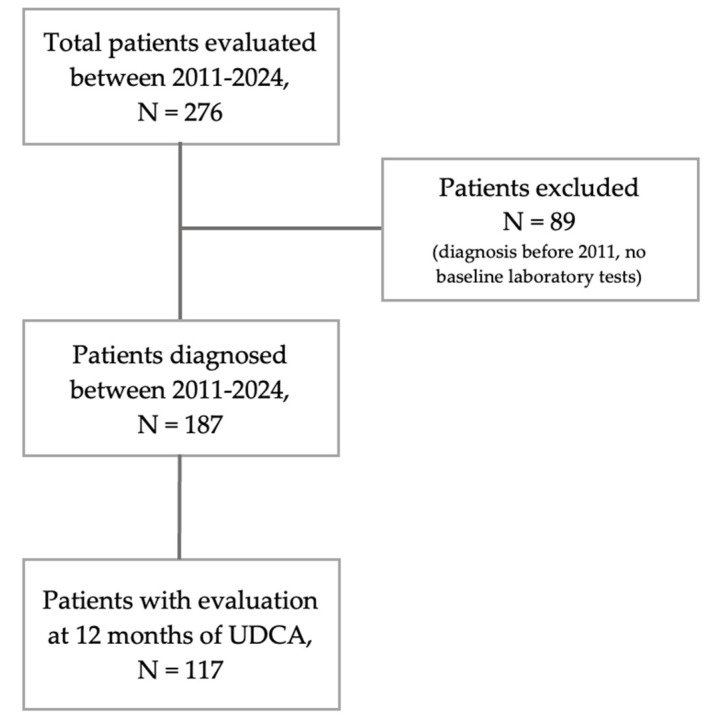
Flowchart of study cohort.

The study involved 276 patients who had at least one evaluation during the study period. The average age at diagnosis was 53.89 years, with women making up 95.28% (female to male ratio 19:1). The median follow-up period for LRO was 96.54 months (95% confidence interval [CI] = 87.27–105.81). Figure 1 presents a visual breakdown of the patients included in the study.

Of the 276 patients evaluated for clinical associations and long-term LRO, 89 were excluded due to a diagnosis before 2011 or the lack of baseline lab data. Therefore, 187 patients were enrolled, diagnosed between 2011 and 2024, and had baseline lab data. Among these, 117 were evaluated at 12 months after starting treatment with UDCA, and 140 were assessed at 24 months. 98 of the patients evaluated at 12 months were also evaluated at 24 months. It should be noted that those evaluated at 24 months were diagnosed from 2011 to 2023.

Table 1 compares the entire group of patients with those diagnosed between 2011 and 2024. It is observed that the statistical differences between the total patient group and the subgroup diagnosed between 2011–2024 are reflected in the follow-up period, age at diagnosis (which is significantly higher for patients in the subgroup), and the presence of liver cirrhosis.

In Table 2, we present the baseline laboratory tests for patients diagnosed between 2011 and 2024, as shown in Figure 1.

We have observed that the proportion of women remains high at 94.79%. ALP had a mean value of 278 U/L, and 78.12% of patients had levels above the ULN of 120 U/L. In contrast, GGT had a mean value of 261 U/L, with 71.35% of patients having levels above the ULN of 55 U/L. It is important to note that 34.75% of patients had a higher GGT/ULN level than the ALP/ULN level, based on logarithmic calculation.

Additionally, 56.2% of patients had total cholesterol levels above the upper normal limit (ULN) of 200 mg/dL, with a mean value of 221 mg/dL, and 16.6% had total triglyceride levels above the ULN of 150 mg/dL, with a mean of 113.9 mg/dL. During follow-up, the mean total cholesterol decreased to 209.33 mg/dL, and the mean triglyceride level dropped to 110.82 mg/dL, with these differences being statistically significant (*p* = 0.007).

### 3.2. Special Populations

The patients diagnosed with AIH-PBC Variant Syndrome made up 25% (N = 69) of the entire study population, which was more common in patients without liver cirrhosis. PSC Variant Syndrome was less frequent and didn’t show a significant difference between cirrhotic and non-cirrhotic patients (3.57% versus 3.05%, *p* = 0.810). However, it is noteworthy that nine patients exhibited a Variant Syndrome between PBC and PSC, which is an entity rarely described in the literature. These patients had PSC without associated inflammatory bowel disease (IBD); four also had autoimmune hepatitis (AIH).

In four patients, liver cirrhosis developed as a progression of their disease. Notably, all patients with the PBC-PSC phenotype were female.

The presence of AMA M2 antibodies was reported in 85.51% of patients, and specific ANA antibodies sp100 and gp210 were found in 7.61% and 7.25% of cases, respectively. Liver cirrhosis was observed in 40.58% of cases, with 18.48% having decompensated cirrhosis, most commonly presenting as jaundice and ascites.

Liver-related death was reported in 4.35% of patients. Considering the types of positive antibodies, no statistically significant differences were observed in the occurrence of liver cirrhosis, liver-related death, liver transplant, or complications related to liver cirrhosis.

### 3.3. Response to Treatment Analysis

To evaluate the response criteria at 12 months of UDCA treatment, a comparative analysis was conducted with the Paris II criteria. This analysis examined patients who responded to the POISE criteria and the Barcelona criteria, as well as their respective responses to the Paris II criteria. Furthermore, we analysed biochemical cholestasis markers, including GGT and ALP, with varying cut-offs to define cholestasis. Table 3 indicates that 44.44% of the patients were respondents at 12 months, according to the Paris II criteria. 41.77% responded to POISE (78.85% of whom also responded to Paris II), and 52.14% responded to Barcelona (71.15% of whom also responded to Paris II).

Among the biochemical cholestasis markers, the decrease in ALP <1.67 × ULN (62.39%) and the reduction in GGT <1.67 × ULN (59.82%) included the majority of patients, also correlating with the Paris II criteria.

The differences between these criteria were statistically significant. Using the specified cut-offs of 0.3 for the GLOBE Score and 1.41 for the URS for response to UDCA treatment, we found that 67.52% of patients responded according to the URS, with 100% of those responding to Paris II and 52.99% responding according to the GLOBE score cut-off.

It should be noted that the recommended doses of UDCA were recorded in the patients’ files, ranging from 750 mg to 1500 mg per day, but without data on body weight (necessary to determine if the dose was correct). Regarding intolerance to UDCA treatment, this was reported in three cases.

**Figure 2:**
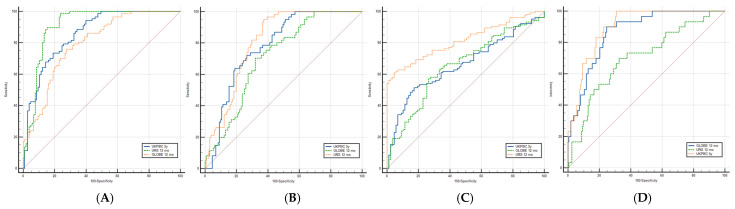
ROC Curves for the predictive capacity of GLOBE score at follow-up, UK-PBC 5 years, and URS at follow-up for responders according to Paris II criteria (**A**), POISE criteria (**B**), and Barcelona criteria (**C**). In (**D**), the outcome is LRO.

To illustrate and highlight the performance of the different prognostic scores used in the data analysis, an analysis using ROC curves was performed for the GLOBE, UK-PBC, and URS scores. This is shown in Figure 2. Each of the figures represents the analysis of the predictive capacity of the responding patients according to the Paris II (Figure 2A), POISE (Figure 2B), and Barcelona (Figure 2C) criteria, as well as the composite outcome LRO.

The results have been described in detail in Table 4, where the areas under ROC (AUROC) are presented, with the UK-PBC at 5 years having the best results for Paris II (AUROC 0.859, Sensitivity = 99%; Specificity = 98.9%) and liver-related outcomes (AUROC 0.899; Sensitivity = 96.7%; Specificity = 30.9%), GLOBE score for liver-related outcomes (AUROC 0.867; Sensitivity = 96.7%; Specificity = 53.7%).

In Table 5, we performed a univariate analysis of potential predictor variables for UDCA response according to the Paris II criteria at 12 months. It is observed that predictive factors include higher age at diagnosis, lower ALP (mean ALP in non-responders 411.69 ± 291.58), as well as lower TB, ALT, AST, and total albumin at diagnosis. The presence of Sp100 antibodies at diagnosis was associated with the response to UDCA. A lower FIB4 index and APRI clinical fibrosis scores correlated with treatment response. In the multivariate logistic regression analysis, ALP was identified as an independent predictor of response to UDCA treatment.

**Figure 3:**
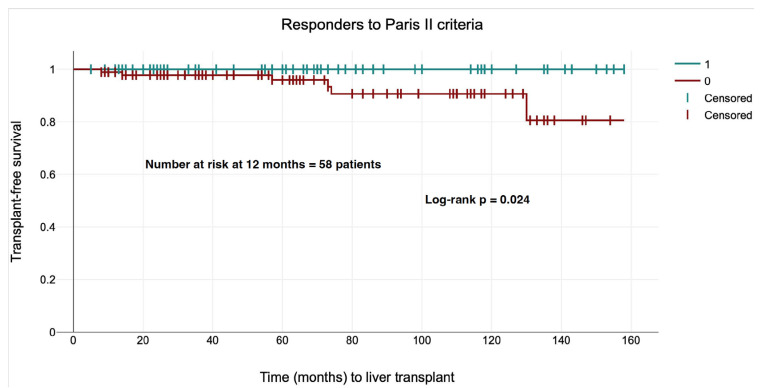
Survival curve analysis for transplant-free survival adjusted to responders to the Paris II criteria. The number of at-risk patients at 12 months was calculated from non-responders (N = 58, 49.57% of patients).

The liver transplant-free survival analysis, presented in Figure 3 using the Kaplan–Meier method, demonstrated a significant disparity between patients who exhibited a response to UDCA treatment, as defined by the Paris II criteria, and those who did not. Within the group of responders, no adverse events (liver transplantation) were observed. The Log-Rank test (chi-square = 5.11, *p* = 0.024) revealed a statistically significant association between UDCA response and improved prognosis in terms of survival without liver transplantation.

Subsequently, we aimed to evaluate the response to treatment at 24 months, based on the distribution of patients into subgroups, as shown in Table 6, to capture the dynamics of response to UDCA in our population.

The Barcelona criteria included more patients than the Paris II and POISE. It is noted that there were no statistically significant differences in response rates between the two subgroups. The comparison of responses at 12 and 24 months (sub-group A) showed that the response rates were significantly higher at 24 months of treatment (*p* < 0.001) for the three criteria analyzed.

## 4. Discussion

This retrospective single-centre study on patients diagnosed with PBC in Romania is one of the first of its kind in the country. The study aimed to assess patients’ responses to first-line treatment, specifically UDCA, the sole available treatment option at the time of this study. The medical centre conducting the study is a high-volume centre for liver diseases and liver transplantation in Romania, concentrating a substantial number of patients and complex cases. However, this large sample size may also influence the profile of these patients, potentially leading to the inclusion of those with more severe and advanced forms of the disease.

It is very important to describe the population of patients with PBC to stratify their risk, response to treatment, and long-term evolution. The female-to-male ratio reported in our population was among the highest ratios recorded compared to other studies in Europe, where ratios of 80–90% were observed, as well as in Asia, where the proportion of male patients is higher [4,10,30]. The presence of liver cirrhosis in the study population is an important factor in determining the prognosis, given that PBC is a progressive disease. It should be noted that the data from the study refer to the presence of liver cirrhosis in the evolution of patients, not only at the time of diagnosis, thus being comparable with other studies conducted previously [23,24,26]. This high rate of cirrhosis is due to the fact that the study was conducted in a liver transplant center and had a long follow-up period.

PBC can be associated with other autoimmune diseases, and in our study, this was AIH, followed by autoimmune thyroiditis. In comparison, a review showed that patients with PBC most frequently associate with Sjogren’s syndrome, in up to 66% of cases, with Variant Syndromes reported in 5–15%, and autoimmune thyroiditis in up to 15% of cases [10,14]. In the study, a more frequent association with thyroid disease was observed in patients without liver cirrhosis, although this aspect did not impact the evolution of PBC [2,11]. Variant Syndrome with AIH can occur at the time of PBC diagnosis or during its evolution, being more frequent than variant syndrome with PSC. Overlap syndrome with AIH can have an unfavourable impact on the progression of liver disease if not recognised [22,31]. The Variant Syndrome with PSC is a more recently described entity in the literature. Several case series or case presentations have been reported, indicating visible changes on the MRCP in the bile ducts associated with PBC-specific antibodies [12,32]. This phenotype was observed in a small population of patients, with no significant differences between those with favourable and unfavourable outcomes. Unlike the Variant Syndrome with AIH, the one with PSC benefits from the same treatment, UDCA.

To effectively manage patients, it is essential to address their profile, evaluate symptoms (particularly pruritus and fatigue), and perform risk stratification to recommend an integrated treatment approach [1,15,33]. While all patients in the study population received UDCA as the initial treatment option, the tolerance profile wasn’t noted in all files of the included patients. A study by Kowdley et al. states that the overall rate of intolerance to UDCA is low, at 3–5%, which is considered insignificant [34].

Additionally, the data on the pharmaceutical forms of the drugs, the time of day treatment was administered, adherence to treatment, and interruptions due to pharmacy unavailability in Romania were not evaluated. These factors could greatly impact the treatment response [10,27,33].

To assess the response to treatment, multiple criteria were considered, based on retrospective databases to predict liver-related outcomes, most commonly defined as death and liver transplantation [24]. In the conducted study, we developed a composite hard outcome measure that encompassed liver transplant, liver-related death, and decompensated cirrhosis events such as variceal bleeding, hepatic encephalopathy, refractory ascites, or jaundice. The defined LRO was employed to compare with established criteria sets, including the Paris II, Barcelona, POISE, URS, UK-PBC, and GLOBE scores. These criteria were selected based on the availability of the corresponding datasets. Composite endpoints in PBC have been previously used to assess patients’ long-term progression, as stated by Gazda et al., in a meta-analysis defining them as involving events like decompensated cirrhosis, liver transplantation, or liver-related death [35]. The COBALT study also evaluated a composite outcome using death, liver transplantation, and liver cirrhosis decompensation to measure the response to obeticholic acid treatment [17].

The application of response criteria has become a recommended tool for stratifying patients with unfavourable disease progression, enabling more meticulous monitoring and inclusion in liver transplant lists. These scores and criteria are user-friendly, whether accessed as variations of biochemical markers or through readily available online calculators. Some of these criteria have been validated across multiple populations in Europe, Asia, and the United States, although data on Eastern Europe is less extensive [23,24,36]. In our study, it was demonstrated that the Paris II criteria correlate most closely with adverse events and decreased cholestasis enzymes.

Given the specific patient profile of our study, characterised by female predominance, limited progression to liver cirrhosis, and frequent association with Variant Syndrome PBC-AIH, as well as PBC-PSC, the classification of patients based on these criteria and the LRO presents an opportunity to evaluate these patients and determine the most appropriate criterion for future studies.

Recent studies on second-line therapy in PBC used the criteria from the study by Nevens et al., which are considered more tolerant of treatment response. However, fewer patients met these criteria at both 12 and 24 months compared to the Barcelona criteria, which included a larger number of patients [16,37,38]. This suggests that evaluating patients over a longer period and selecting criteria based on the population may be necessary [39]. While most studies consider the therapeutic response at 12 months, assessing at 24 months is also important because it demonstrates sustained efficacy and relates to clinical progression. Our study demonstrated an improvement in response rate when assessed at 2 years. This evaluation method was also used in the BEZURSO and POISE studies to determine the effectiveness of second-line drugs [16,35,37,40].

To establish the prognosis in PBC, several scoring systems have been introduced. These scores demonstrated utility in predicting the risk of end-stage liver disease, liver-related mortality, or liver transplantation, potentially exhibiting greater accuracy than the binary criteria for treatment response [24,27,36].

In previous studies, specific cut-offs were specified for the GLOBE score (<0.3) and the URS score (≥1.41), which enable the dichotomization of patients based on their higher or lower risk of complications [19,26,27,28].

Choosing an optimal cut-off for lab tests and continuous scores is usually done by maximizing sensitivity and specificity, using the Youden index, as has been done for continuous URS and GLOBE scores in previous studies [24,25,28]. Another method is to evaluate discriminatory ability using AUROC. This approach emphasizes maximizing true negative and true positive rates, as we did to compare the performance of continuous scores and treatment response criteria [24,26,41]. Another approach to selecting an optimal cut-off involves examining its association with the prediction of a known clinical endpoint (such as liver transplantation or death), as used in the URS study, to identify which ALP decrease had the greatest discriminatory ability [11,28]. This method was also tested by using LRO, against which we evaluated the discriminatory abilities of the scores.

In the study, we observed a strong correlation between the risk of adverse events and the response to treatment, as defined by the Paris II criteria. To facilitate comparative analysis, considering treatment criteria and LRO, the ROC curves demonstrated satisfactory performance for continuous scores, particularly in biochemical response to UDCA and adverse events. These findings are comparable to the index data that used the same thresholds, showing AUROC values between 0.830 and 0.870 for URS and 0.820–0.910 for GLOBE score [23,25,28]. The 5-year UK-PBC score demonstrated predictive capacity, aligning with results from previous studies [23,26]. These data are valuable for stratifying patients based on liver-related events.

In current clinical practice, however, the treatment criteria and variations in laboratory parameters remain the easiest to use, focusing on the decrease in GGT and ALP [10]. Significant data were identified for both the reduction in ALP and GGT. The cut-off of 1.67 x ULN fell into the category of biochemical response to UDCA, being consistent with Paris II criteria. These data demonstrate the usefulness of both the decrease in ALP and GGT in establishing the prognosis in PBC. Some patients may have higher GGT values at diagnosis, a particularity identified in the studied population [19,42]. In addition to the prognostic role of the dynamics of biochemical cholestasis markers, other factors that may influence the prediction of response to UDCA have been observed in other studies. The study by Zhu et al. found higher total cholesterol to be associated with non-response [41]. AST and ALT or TB levels are used in prognostic scores such as URS or GLOBE, with significance also noted in our study [27,28]. Among the clinical factors, autoantibodies play an interesting role in patient diagnosis. The article by Trivella et al. notes that the presence of anti-gp210 is linked to a more aggressive disease, a connection not observed in the study population [14]. Conversely, anti-sp100 was associated with a positive response to treatment, contrasting with data from Martini et al., who report it being linked to a higher risk [11].

The strengths of this study include presenting essential data on a population of patients with PBC in Romania, evaluating their response to the first line of treatment over a long period, at 12 and 24 months. We analyzed different criteria that can be used in future studies. The phenotype of patients with PBC from a Romanian population was described, along with details about the features of specific subpopulations.

The limitations of this study were due to the retrospective data collection, which was inconsistently reported in patient records. There was variability in the assessment and follow-up intervals, treatment tolerability, and both biochemical and clinical responses. This variability increases the risk of bias and limits the generalizability of the results [27]. The risk of bias includes selection bias, as patients treated at the center that enrolls the most patients may have more complex disease, potentially higher compliance, which could overestimate treatment response or prognostic accuracy [27,31]. The study may also show variability in diagnosis and management, as noted by Graf et al. [31]. Patient identification in retrospective studies is another concern; if ICD codes are used, they may be misapplied for other diseases, like PSC [43]. Additionally, there is a risk of bias in accurately assessing treatment response due to confounding factors that cannot be controlled [44]. Regarding the generalizability of information, the monocentric nature of our study limits its applicability due to differences in genetic background, healthcare access, and clinical presentation [26,30]. However, the center is the primary center in Romania that evaluates cholestatic liver diseases and handles liver transplantation in these cases. Extending this study to the main medical centers in Romania would represent a better understanding of the PBC population and response to treatment.

## 5. Conclusions

Our study, conducted on a Romanian population, may contribute to a better understanding of the type of patient who will benefit most from a second-line treatment.

UDCA, as a first-line treatment, has significantly improved the natural history of PBC. However, a substantial portion of the population experiences an insufficient response at 12 months, which is associated with long-term disease progression outcomes.

The factors contributing to the insufficient response to UDCA include the specific characteristics of the population, a lower age at diagnosis, higher ALP values at diagnosis, the absence of sp100 antibodies, and higher clinical fibrosis scores.

## Figures and Tables

**Table 1:** Patient’s baseline characteristics, divided into two subgroups by timeline of diagnosis.

	Total PBC Patients (N = 276)	PBC Diagnosed Between 2011–2024 (N = 187)	*p* Value
**PBC-AIH, %**	25%	23.22%	0.219
**PBC-PSC, %**	3.26%	3.26%	0.086
**Age at diagnosis, mean (CI95%)**	53.89 (52.43–55.34)	55.54 (53.43–56.58)	0.001
**Follow-up period, months (CI95%)**	96.54 (87.37–105.7)	65.6 (59.96–72.56)	<0.001
**Female patients, %**	95.28%	94.11%	0.167
**Bone disease, %**	13.77%	12.8%	0.399
**Thyroid disease, %**	19.57%	18.96%	0.646
**Sjogren’s syndrome, %**	6.88%	7.11%	0.790
**Type 2 DM, %**	5.8%	6.16%	0.641
**Liver transplant, %**	4.35%	3.79%	0.414
**AMA pos, %**	85.51%	87.68%	0.134
**gp210 pos, %**	7.25%	8.53%	0.138
**sp100 pos, %**	7.61%	9%	0.115
**Liver cirrhosis, %**	40.58%	36.02%	0.001
**Decompensated cirrhosis, %**	18.48%	17.54%	0.414
**Ascites, %**	24.11%	27.63%	0.164
**Variceal bleeding, %**	15.18%	19.73%	0.790
**Encephalopathy, %**	8.04%	9.21%	0.924
**Jaundice, %**	28.57%	32.89%	0.668
**Liver-related death, %**	4.35%	4.74%	0.566

**Table 2:** Baseline laboratory values, represented as means (IQR); N = 187 patients.

Variable	Value
**PLT, ×10^9^/L (IQR)**	246.6 (171–299)
**AST, U/L (IQR)**	69.85 (34–83)
**ALT, U/L (IQR)**	93.64 (38–99)
**GGT, U/L (IQR)**	270.2 (68–330)
**ALP, U/L (IQR)**	284.01 (130–362)
**Total bilirubin, mg/dL (IQR)**	1.37 (0.5–1.2)
**Total Cholesterol, mg/dL (IQR)**	222.1 (169–256)
**Total Triglycerides, mg/dL (IQR)**	115.1 (74–131)
**Albumin, mg/dL (IQR)**	4.23 (4.15–4.4)
**GGT/ULN > ALP/ULN, %**	34.75%

**Table 3:** Criteria and quantitative scores evaluated for response to treatment at 12 months of UDCA. *p*-value calculated for the differences between responders and non-responders according to Paris II criteria; N = 117.

Criteria/Score	Patients	Responders to Paris II at 1 year (N = 52)	*p* Value
Paris II, N (%)	52 (44.44)	-	-
POISE, N (%)	49 (41.88)	52 (78.85)	<0.001
Barcelona, N (%)	61 (52.14)	37 (71.15)	<0.001
GGT decrease <1.5 × ULN, N (%)	66 (56.41)	41 (78.84)	<0.001
ALP decrease <1.5 × ULN, N (%)	64 (54.70)	52 (100)	<0.001
GGT decrease <1.67 × ULN, N (%)	70 (59.82)	44 (84.61)	<0.001
ALP decrease <1.67 × ULN, N (%)	73 (62.39)	52 (100)	<0.001
>50% ALP decrease, N (%)	31 (26.49)	16 (30.76)	<0.001
>40% ALP decrease, N (%)	47 (40.17)	24 (46.15)	0.391
GLOBE score < 0.3, N (%)	62 (52.99)	40 (76.92)	<0.001
URS ≥ 1.41, N (%)	79 (67.52)	52 (100)	<0.001

**Table 4:** Area under the Receiver Operating Characteristic (AUROC) curve (with sensitivity-Ss and specificity-Sp) for quantitative prognosis scores at follow-up (12 months), for prediction of responders according to Paris II criteria, POISE criteria, Barcelona criteria, and for LRO.

Score/Criteria	Paris II (Responders = 52)	POISE (Responders = 49)	Barcelona (Responders = 61)	Liver-Related Outcomes (N = 30)
**UK-PBC 5 years, AUROC (CI 95%)**	0.859 (Ss =9 9%; Sp = 98.9%)	0.782 (Ss = 95.4%; Sp = 98.4%)	0.662 (Ss = 98.9%; Sp = 99%)	0.899 (Ss = 96.7%; Sp = 30.9%)
**GLOBE score at follow-up, AUROC (CI 95%)**	0.797 (Ss = 99%; Sp = 82.8%)	0.715 (Ss = 99.2%; Sp = 95.1%)	0.655 (Ss = 98.9%; Sp = 99%)	0.867 (Ss = 96.7%; Sp = 53.7%)
**URS at follow-up, AUROC (CI 95%)**	0.910 (Ss = 98.9%; Sp = 29.5%)	0.811 (Ss = 98.4%; Sp = 46.6%)	0.816 (Ss = 99%; Sp = 94.3%)	0.702 (Ss = 99.4%; Sp = 96.7%)

**Table 5:** Univariate analysis and multivariate analysis (using logistic regression) for predictors of response to Paris II criteria at 12 months. Values are represented as Mean ± SD, or Chi-square (X^2^) in univariate analysis.

	Univariate Analysis		Multivariate Analysis		
Variable	Mean ± SD	*p* Value	OR	CI 95%	*p* Value
**Age**	58.52 ± 11.88	0.032	1.02	0.97–1.07	0.406
**GGT**	226.29 ± 246.64	0.090	-	-	-
**ALP**	231.81 ± 177.88	<0.001	1.00	0.99–1.00	<0.001
**TB**	0.78 ± 0.56	<0.001	0.53	0.25–1.10	0.089
**ALT**	78.23 ± 64	0.032	0.99	0.98–1.01	0.324
**AST**	59.38 ± 50.26	0.014	1.01	0.99–1.04	0.258
**ALB**	4.26 ± 0.56	0.003	1.96	0.70–5.52	0.200
**PLT**	255.13 ± 92.76	0.136	-	-	-
**Cholesterol**	201.82 ± 57.65	0.120	-	-	-
**Triglycerides**	114.19 ± 47.9	0.549	-	-	-
**AMA-M2 presence**	0.23(X^2^)	0.630	-	-	-
**sp100 presence**	3.93(X^2^)	0.047	2.40	0.46–12.38	0.297
**gp210 presence**	1.11(X^2^)	0.293	-	-	-
**FIB4 index**	1.95 ± 2.03	0.013	1.11	0.51–2.41	0.795
**APRI score**	0.79 ± 0.9	0.004	0.49	0.05–4.95	0.548

**Table 6:** Presents data on the response to UDCA at 24 months across patient subgroups, evaluated at baseline (0), 12, and 24 months, and at baseline and 24 months, respectively.

	Patients Evaluated at 0–12–24 Months, N = 98 (A)	Patients Evaluated at Baseline and 24 months, N = 140 (B)	*p* Value
Paris II, %	55.10%	56.42%	0.427
Barcelona, %	66.33%	70%	0.096
POISE, %	47.96%	45.71%	0.441

## Data Availability

The data on which this study is based will be made available upon request.

## Abbreviations

The following abbreviations are used in this manuscript:

PBC	Primary Biliary Cholangitis
ALP	Alkaline Phosphatase
GGT	Gamma-Glutamyl-Transpeptidase
AMA-M2	Antimitochondrial Antibody M2
UDCA	Ursodeoxycholic Acid
ULN	Upper Limit of Normal
Paris II	Paris II Response Criteria
POISE	POISE Response Criteria/Trial
GLOBE	Global Assessment of Liver Outcomes Score for PBC
UK-PBC	United Kingdom Primary Biliary Cholangitis Score
URS	UDCA Response Score
APRI	AST to PLT Ratio Index
FIB-4	Fibrosis-4 index
ROC	Receiver Operating Characteristic
AUROC	Area Under the Receiver Operating Characteristic Curve
CI	Confidence Interval
EASL	European Association for the Study of the Liver
AMA	Antimitochondrial Antibody
ANAs	Antinuclear Autoantibodies
gp210	Glycoprotein 210
sp100	Nuclear Body Protein sp100
US	United States
PSC	Primary Sclerosing Cholangitis
MRCP	Magnetic Resonance Cholangiopancreatography
AIH	Autoimmune Hepatitis
IgG	Immunoglobulin G
AST	Aspartate Aminotransferase
OCA	Obeticholic Acid
FXR	Farnesoid X Receptor
FDA	Food and Drug Administration
EMA	European Medicines Agency
PPAR	Peroxisome Proliferator-Activated Receptor
TB	Total Bilirubin
ALT	Alanine Aminotransferase
ALB	Albumin
PLT	Platelet Count
LLN	Lower Limit of Normal
OR	Odds Ratio
SD	Standard Deviation
IQR	Interquartile Range
LRO	Liver-Related Outcomes
